# Irritation from metalwork after ankle arthrodesis fixed using screws: a proportional meta-analysis and systematic review

**DOI:** 10.1007/s00402-023-04813-1

**Published:** 2023-02-16

**Authors:** Antonio Izzo, Arianna Sgadari, Salvatore Santagata, Antonio Coviello, Andrea Cozzolino, Massimo Mariconda, Alessio Bernasconi

**Affiliations:** Department of Public Health, Trauma and Orthopaedics, University of Naples Federico II, Naples, Italy

**Keywords:** Ankle, Arthrodesis, Fusion, Screws, Fibular graft, Union

## Abstract

**Objective:**

Ankle arthrodesis (AA) is often fixed using cannulated screws. The irritation from metalwork is a relatively common complication, but there is no consensus regarding the need to remove the screws on a systematic basis. The aim of this study was to determine (1) the proportion of screws removed after AA and (2) whether predictors of screw removal could be identified.

**Methods:**

This PRISMA-compliant systematic review was part of a larger previous protocol registered on the PROSPERO platform. Multiple databases were searched including studies in which patients undergone AA using screws as exclusive fixation method were followed. Data were harvested regarding the cohort, the study design, the surgical technique, the nonunion and complication rate at the longest follow-up. Risk of bias was assessed using the modified Coleman Methodology Score (mCMS).

**Results:**

Forty-four series of patients from thirty-eight studies (1990 ankles, 1934 patients) were selected. The average follow-up was 40.8 months (range 12–110). In all studies, hardware was removed due to symptoms reported by patients and related to the screws. The pooled proportion of removal of metalwork was 3% (95% CI 2–4). The pooled proportion of fusion was 96% (95%CI 95–98), while the pooled proportion of complications and reoperations (excluding the removal of metalwork) stood at 15% (95% CI 11–18) and 3% (95% CI 2–4), respectively. The mean mCMS (50.8 ± 8.1, range 35–66) revealed only an overall fair quality of studies. The univariate analysis and the multivariate model showed that the year of publication (*R* = − 0.004; *p* = 0.01) and the number of screws (*R* = 0.08; *p* = 0.01) were associated with the screw removal rate. Specifically, we found that over time the removal rate decreased by 0.4% per year and that the use of three screws instead of two reduced the risk of removal of metalwork by 8%.

**Conclusions:**

In this review, removal of metalwork after ankle arthrodesis using cannulated screws was needed in 3% of cases at an average follow-up of 40.8 months. It was indicated only in case of symptoms related to soft tissue irritation from screws. The use of three screws was paradoxically related to a reduced risk of removal of screws as compared to two-screw constructs.

**Level of evidence:**

Level IV, systematic review of Level IV.

## Introduction

Ankle Arthrodesis (AA) is a reliable option to treat end-stage ankle osteoarthritis resistant to conservative approaches [1–6]. To date, ankle replacement is gaining space in the foot and ankle field with new implants showing improved survival rates as compared to a few years ago, nevertheless AA remains the main choice in patients with history of ankle infection, significant tibiotalar deformity, poor bone quality or other contraindication to joint replacement [1–3]. Multiple fixation methods are available to stabilize the tibiotalar joint (screws, plates, external fixators or a combination of them) [2, 7–11] with no evidence of superiority of a method over one other. Among these methods, cannulated screws can be used in different number (two, three or less frequently more) and configuration (parallel or crossed) [12–15]. Furthermore, the use of a lateral fibular graft may offer additional biological and mechanical support to the arthrodesis [13], being therefore advocated by some authors.

Once the fusion has been achieved, the metalwork may be either removed or left in place. On a side, the systematic removal of screws may help reduce the risk of soft-tissue irritation at the price of risking further complications due to a second operation (e.g., infection, intraoperative fracture, nerve injury, etc.). On the other side, it may be suggested that the screws should be left in place and removed only in case of pain or discomfort reported by the patient. To date, there is no consensus in this field, therefore surgeons will generally make decisions based on their personal experience rather than on clear evidence.

In this study, we aimed to review the current literature to determine (1) the weighted proportion of screws removed after AA and (2) whether predictors of screw removal could be identified. Based on common experience, we hypothesized that the risk of removal of screws would be low and that a greater number of screws would lead to a higher risk of metalwork irritation.

## Methods

### Protocol and registration

This systematic review followed the Preferred Reporting Items for Systematic reviews and Meta-Analyses (PRISMA) statement and was part of a larger protocol prospectively registered in the PROSPERO database (CRD42022322784).

### Eligibility criteria

The inclusion criteria were as follows: studies reporting data after AA (open or arthroscopically assisted) stabilized using only screws in patients aged between 18 and 85 years; clear description of the surgical technique with one or more statements about the number of screws used; studies including a sample size larger than 10 ankles; assessment of radiographic results through pre- and postoperative weightbearing standardized radiographs; reporting complications and reoperations after AA; minimum follow-up of 12 months; randomized, quasi-randomized, prospective and retrospective cohort studies, case series, technical notes; published in English; full text availability either online either after direct contact with the authors.

Exclusion criteria were the following: studies reporting results after AA stabilized using other methods (nail, external fixator, plate, hybrid constructs); data on skeletally immature patients; case reports, biomechanical studies, cadaveric studies, expert opinions, letters to the editor, studies on animals and instructional courses. Narrative or systematic reviews were also excluded from this study, but references were double checked to identify potential eligible studies.

### Information sources and search

Pubmed, Embase, Cochrane Library and Scopus databases were searched from the earliest entries through November 20, 2022 with the following key words and Boolean operators: ((ankle) AND (arthrodes*)) OR ((ankle) AND (fusion)). Additional studies were identified in the bibliographies of articles. Two reviewers (AI and SS) independently screened the results of the research, then the full text of eligible studies was analyzed. Disputes were resolved by the senior author (AB). Unpublished studies and gray literature were not considered.

### Data charting and items

Data were charted independently by two investigators (AI and AS) using an Excel sheet. Data were harvested regarding the cohort, the study design, the surgical technique and the outcome after the procedure. The primary outcome of this study was the need of removal of screws at the longest follow-up. The fusion rate, the complication rate and the need of reoperation (excluding the removal of metalwork) were the secondary outcomes.

### Risk of bias

The modified Coleman Methodology Score (mCMS) was used to assess the quality of studies included, as in previous foot and ankle literature [16, 17], ranging from 0 to 100. Two investigators performed the mCMS assessment twice (AI and AC), with an interval of 10 days, then discussed the scores when more than a two-point difference was present, until consensus was reached. A score higher than 85 was considered excellent, good from 70 to 84, moderate from 50 to 69 and poor when less than 50 [16, 17].

### Synthesis of results

Baseline data were reported as average value, standard deviation (SD) or 95% confidence interval (95% CI) and range values (minimum and maximum). A proportional meta-analysis was run to pool data regarding the rate of screw removal, fusion, nonunion, complication and reoperation. The ‘metaprop’ command was used to compute 95% CI using the score statistic and the exact binomial method and incorporate the Freeman-Tukey double arcsine transformation of proportions. Heterogeneity among studies was assessed through the Higgins’ *I*^2^ statistic and a random-effect model was applied in all cases.

Univariate linear regression was run to test demographics (sample size, sex, age), characteristics of the study (year of publication, mCMS, Level of Evidence (LoE) and length of follow-up) and type of surgery (arthroscopic or open procedure, number of screws used, configuration of screws, use of graft) against the need of screw removal. The association between variables (considering a continuous dependent variable) was tested through Pearson’s coefficient correlation (for continuous independent variables) and Wilcoxon rank-sum test (for categorical independent variables). For categorical independent variables where more than two categories were expected a Kruskal–Wallis test was used. A multivariate model was then used including all those variables significantly associated to the removal rate at the univariate analysis. Dummy variables were generated to handle categorical variables in the regression analysis. Parameters with *P* values < 0.05 were considered statistically significant in the final model. All analyses were performed using STATA statistical software package (Version 16.0, StataCorp, 2019).

## Results

**Fig. 1:**
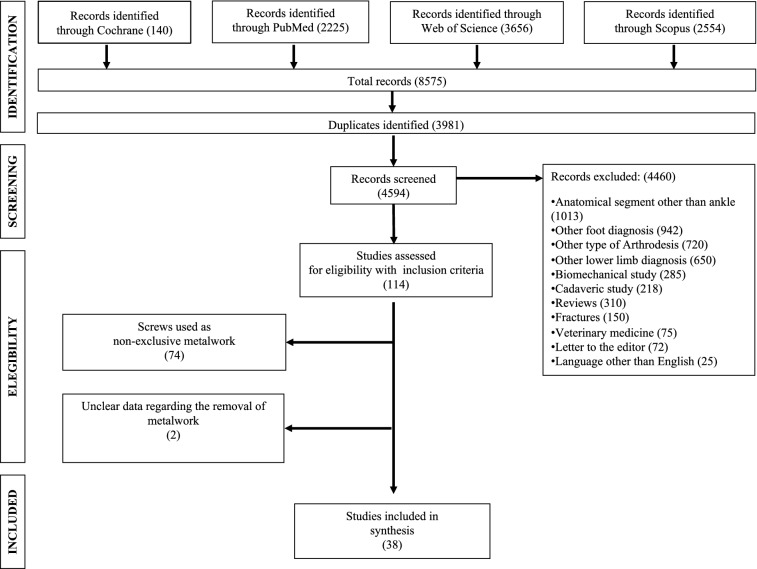
Flow chart for studies included in this systematic review

**Fig. 2:**
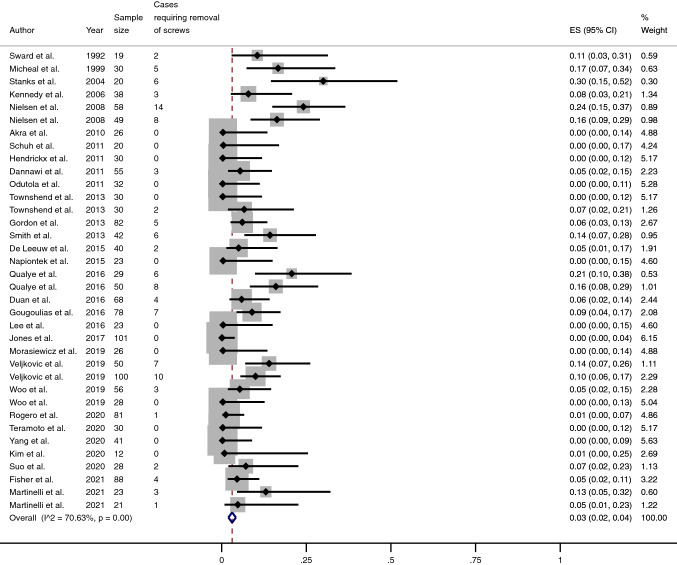
Meta-analysis of the proportion of removal of screws in patients undergone Ankle Arthrodesis fixed using cannulated screws. Output generated by the Stata procedure *metaprop*

Forty-four series of patients from thirty-eight studies (1990 ankles, 1934 patients) were selected (Tables 1 and 2) (Fig. 1) [1, 3, 5, 12, 15, 18–50]. The average follow-up was 40.8 months (range 12–110). In all studies, hardware was removed due to symptoms reported by patients and related to the screws. The non-weighted screw removal rate was 7% (range 0–30), but the pooled proportion of removal of metalwork was 3% (95% CI 2–4) (Fig. 2).

**Table 1:** Main characteristics of studies included in this review

Characteristics of studies included	Mean (SD)	Range (min – max)
Sample size (ankles), *N*	45.2 (26.7)	12–118
Sample size (patients), *N*	43.9 (26.2)	12–116
Age, *y*	56.4 (5.8)	43 -70
Sex, *% female*	42.7 (14)	17–67
mCMS	50.8 (8)	35–66

*mCMS* modified Coleman Methodology Score

**Table 2:** Surgical details from studies included in this review

Surgical details	(%)
Arthroscopic procedure	Y: 47 N: 53
Graft	Y: 16 N: 84
Number of screws	< 3: 79 3: 21
Orientation	Crossed: 43 Parallel: 32 Variable: 25

### Predictors of outcome

**Fig. 3:**
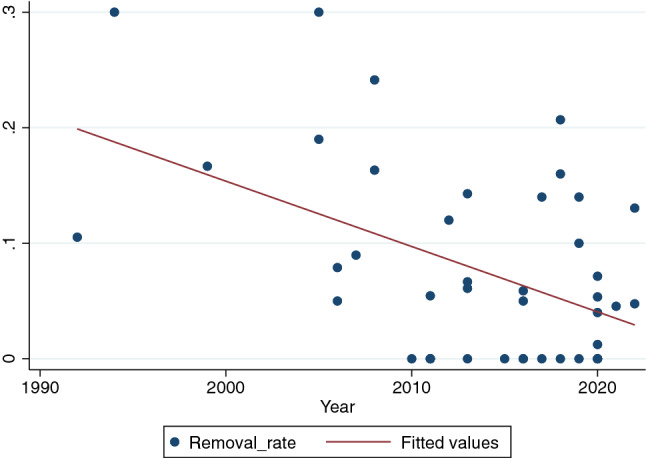
Scatter diagram illustrating the negative correlation between the year of publication of studies included in this review (x axis) and the rate of screw removal reported in each study (y axis, where 0.01 corresponds to 1%) (*R* = − 0.48; *p* =  < 0.001)

The univariate analysis suggested that the rate of removal of screws was associated with the year of publication of the study (*R* = − 0.48; *p* =  < 0.001) and with the number of screws used for the arthrodesis (*p* = 0.004). The multivariate model confirmed that both these variables were significantly associated with the need of screw removal (R = − 0.004; *p* = 0.01 for the year of publication and *R* = 0.08; *p* = 0.01 for the number of screws). Specifically, we found that over time the removal rate decreased by 0.4% per every year passed by (Fig. 3) and that the use of three screws instead of two reduced the risk of removal of metalwork by 8%.

### Secondary outcomes

**Fig. 4:**
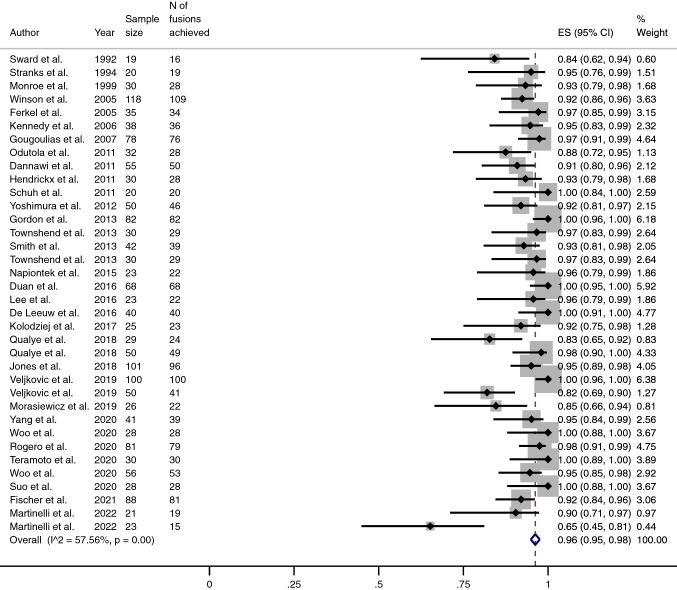
Meta-analysis of the proportion of fusions achieved in patients undergone Ankle Arthrodesis fixed using cannulated screws. Output generated by the Stata procedure *metaprop*

**Fig. 5:**
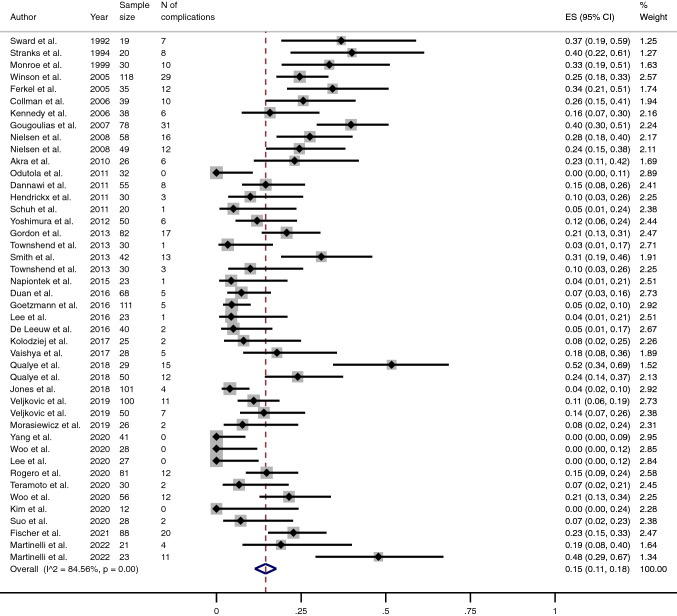
Meta-analysis of the proportion of complications in patients undergone Ankle Arthrodesis fixed using cannulated screws. Output generated by the Stata procedure *metaprop*

**Fig. 6:**
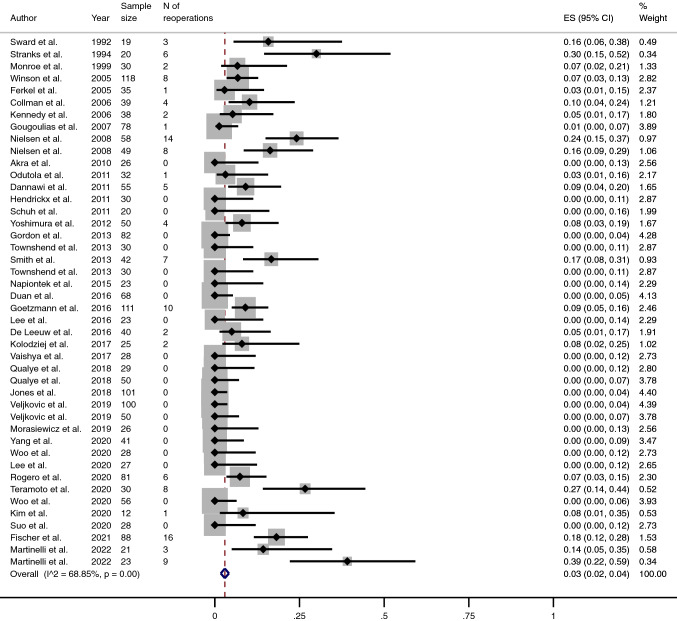
Meta-analysis of the proportion of reoperations (excluding removal of metalwork) in patients undergone Ankle Arthrodesis fixed using cannulated screws. Output generated by the Stata procedure *metaprop*

The pooled proportion of fusion was 96% (95% CI 95–98) (Fig. 4), while the pooled proportion of complications and reoperations (excluding the removal of metalwork) stood at 15% (95% CI 11–18) and 3% (95% CI 2–4) (Table 3) (Figs. 5 and 6), respectively. The mean mCMS (50.8 ± 8.1, range 35–66) revealed only an overall fair quality of studies.

**Table 3:** Main outcomes from studies included in this review, reported both as non-weighted and pooled proportion

Outcome	Non-weighted proportion		Pooled proportion (%)	
	Mean	95% CI	Mean	95% CI
Removal rate	7.6	5–10	5	4–7
Non Union rate	7.1	4.9–9.3	4	3–5
Fusion Rate	92.8	90.6–95	96	95–98
Complication rate	16.9	12.6–21.1	15	11–18
Reoperation rate	8.1	5.1–11	3	2–4

## Discussion

The main finding of this study was that on a cohort of almost 2000 osteoarthritic ankles undergone ankle arthrodesis stabilized using cannulated screws the removal of metalwork was always performed because of soft tissue irritation related to the screws and was finally required in 3% of cases. Excluding the removal of metalwork, the pooled rate of complications was 15% but a second surgery was necessary only in 3% of cases at a 40-month mean follow-up.

Regarding predictors of removal of metalwork, both the univariate and multivariate analysis suggested a negative correlation between the year of publication and the removal of screws, with a reduction in terms of removal rate by 4% every 10 years. This would possibly reflect a reduced risk of irritation from metalwork with the advancement of technology in materials, screw designs and surgical techniques. One could argue that the use of headless screws might play a key role in this scenario, however we’d like to emphasize that out of thirty-eight studies included in this review only two papers by Odutola et al. [30] and Kolodziej et al. [40] reported the use of headless screws [30]. In their papers, the authors have demonstrated that this type of metalwork may reduce the risk of removal at 0% but with a nonunion rate standing at 8–12% which is considered high if compared with other series. Interestingly, the authors have reported a cost of 1285 pounds sterling per every case of metalwork removal in the United Kingdom, which the physician should take into account when discussing this type of procedures.

On a pathophysiological basis, we would have expected to find a correlation between a greater number of screws and a greater risk of removal of metalwork due to the increased total space occupied by the screw heads. We were surprised to see that in this review using three screws instead of two might be a protective element against the risk of removal of metalwork. In our opinion, this could be theoretically due to two reasons: first, the use of two screws may potentially lead to a more frequent use of washers which often do not seat completely on the cortical bone and may possibly lead to irritate surrounding soft tissues; second, the use of a third screw could incentivize the surgeon to place more carefully (and maybe in a more appropriate position) the first two since some room has to be left for the third one. To the best of our knowledge, no other study has analyzed this aspect so far, therefore, a comparison with previous literature was not possible. We advocate that a robust approach taking into accounts potential confounders should be mandatory in future studies to draw conclusions on risk factors for a second surgery after AA.

In our opinion, the final pooled removal rate at 3% represented an average value between a group of studies with greater figures and the fifteen cohorts in which a 0% removal rate was reported [1, 26–28, 30, 31, 35, 37, 39, 40, 42, 43, 45, 47, 48]. Such a low rate probably explains why most surgeons feel that removal of metalwork should not be advised as a routinary procedure. Of note, the pooled proportion of patients requiring removal of metalwork was much lower than the simple non-weighted mathematical average of different studies (7%), which suggests that larger studies tend to report a reduced need to remove the metalwork. On the balance, the relationship between a low risk of irritation from metalwork and all the risks inherently related to surgery leads most surgeons to remove screws only in symptomatic patients. The mean follow-up at 40 months was probably appropriate since in our experience the irritation produced by metalwork generally presents quite early during the first months or years after surgery (except in case of delayed breakage of screws).

Finally, it should be highlighted that, in the majority of studies here included, the conventional follow-up of fused ankles was carried out using standard radiographic imaging, while computed tomography was requested only in selected cases. Due to inherent biases related to radiographs (superimposition of bones, rotation of the source or of the foot, experience of the operator, etc.) [51–56] it would be difficult to extract accurate data about the position of the screw head, the orientation of screws and their entry point as variables potentially related to the risk of soft tissue irritation. In patients complaining of postoperative pain potentially related to metalware irritation the use of recently introduced cone beam weight bearing computed tomography [51–56] could help obtain such information along with data on the fusion of the arthrodesis site and the alignment of the ankle, both important for a correct assessment of the patient during his follow-up.

This study is not without limitations. First, although we included only studies performing ankle arthrodesis using cannulated screws, we acknowledge that the surgical technique adopted by different authors was heterogeneous (in terms of number of screws, metalwork positioning, use of arthroscopy and grafting etc.) which may weaken the strength of our findings. Also, the average quality of studies was only fair as demonstrated by the mCMS and all of them had a retrospective design with a Level of Evidence at III of IV. Third, the removal of metalwork was never considered a primary outcome in the studies selected, which may be considered a potential source of bias.

## Conclusion

In this review, removal of metalwork after ankle arthrodesis using cannulated screws was needed in 3% of cases and was indicated only in case of symptoms related to irritation from metalwork. The pooled fusion rate after ankle arthrodesis using cannulated screws stood at 96%. These data could be useful in clinical practice to counsel patients correctly in the pre-operative setting. We also demonstrated that the need of removal of metalwork is progressively reducing as the time passes by and that using three screws instead of two to fix the tibiotalar fusion site might lead to a reduced risk of metalwork removal. Further studies are needed to confirm or disprove the findings of this review.

## Data Availability

Data of this study can be made available upon request.
